# Differential Cultivation of *Francisella tularensis* Induces Changes in the Immune Response to and Protective Efficacy of Whole Cell-Based Inactivated Vaccines

**DOI:** 10.3389/fimmu.2016.00677

**Published:** 2017-01-10

**Authors:** Sudeep Kumar, Raju Sunagar, Giang Pham, Brian J. Franz, Sarah J. Rosa, Karsten R. O. Hazlett, Edmund J. Gosselin

**Affiliations:** ^1^Center for Immunology and Microbial Diseases, Albany Medical College, Albany, NY, USA

**Keywords:** APC, whole cell-based vaccines, *Francisella tularensis*, costimulatory molecules, growth factors

## Abstract

*Francisella tularensis* (*Ft*) is a category A biothreat agent for which there is no Food and Drug Administration-approved vaccine. *Ft* can survive in a variety of habitats with a remarkable ability to adapt to changing environmental conditions. Furthermore, *Ft* expresses distinct sets of antigens (Ags) when inside of macrophages (its *in vivo* host) as compared to those grown *in vitro* with Mueller Hinton Broth (MHB). However, in contrast to MHB-grown *Ft, Ft* grown in Brain-Heart Infusion (BHI) more closely mimics the antigenic profile of macrophage-grown *Ft*. Thus, we anticipated that when used as a vaccine, BHI-grown *Ft* would provide better protection compared to MHB-grown *Ft*, primarily due to its greater antigenic similarity to *Ft* circulating inside the host (macrophages) during natural infection. Our investigation, however, revealed that inactivated *Ft* (i*Ft*) grown in MHB (i*Ft-*MHB) exhibited superior protective activity when used as a vaccine, as compared to i*Ft* grown in BHI (i*Ft-*BHI). The superior protection afforded by i*Ft*-MHB compared to that of i*Ft*-BHI was associated with significantly lower bacterial burden and inflammation in the lungs and spleens of vaccinated mice. Moreover, i*Ft*-MHB also induced increased levels of *Ft-*specific IgG. Further evaluation of early immunological cues also revealed that i*Ft*-MHB exhibits increased engagement of Ag-presenting cells including increased i*Ft* binding to dendritic cells, increased expression of costimulatory markers, and increased secretion of pro-inflammatory cytokines. Importantly, these studies directly demonstrate that *Ft* growth conditions strongly impact *Ft* vaccine efficacy and that the growth medium used to produce whole cell vaccines to *Ft* must be a key consideration in the development of a tularemia vaccine.

## Introduction

Whole cell-based vaccines are preferred in the absence of well-defined protective immunogens. In addition, whole cell-based vaccines provide a broad range of potential protective antigens (Ags) and immune stimulatory molecules, which can include proteins, polysaccharides, and pathogen-associated molecular pattern molecules (PAMPs). In the absence of an identified protective Ag, directing the immune response toward an array of Ags that the host is likely to encounter during a natural infection maximizes the potential for a protective immune response to be generated upon vaccination. In this regard, the composition of the antigenic repertoire expressed by the constituent whole cell vaccine becomes very important, while PAMPs can provide molecular components required for the activation of Ag-presenting cells (APCs). Furthermore, activation of APCs is a key requirement for the generation of an optimal adaptive immune response against cognate Ags contained within such vaccines.

Importantly, the *in vitro* culture medium utilized to generate whole cell-based vaccines can significantly impact the expression of antigenic determinants and PAMPs on the infectious agent. Several microbes have been shown to differentially express Ags depending on the growth medium utilized, including *Escherichia coli, Salmonella enterica* (1), *Listeria monocytogenes* (2), *Helicobacter pylori* (3), and *Mycobacterium bovis* (BCG) (4, 5). Direct evidence supporting an impact of growth medium on the efficacy of whole cell-based vaccines comes from a study involving BCG where its differential cultivation in Sauton versus Middlebrook medium significantly altered its protective efficacy (6). However, the mechanism responsible for this apparent difference has not been well established.

In regard to *Francisella tularensis* (*Ft*), it has been found that its antigenic repertoire can change depending on the growth medium employed for *in vitro* cultivation. Specifically, we previously demonstrated that the protein profile expressed by Brain-Heart Infusion (BHI)-grown *Ft* closely resembles that of *in vivo* circulating *Ft*, while *Ft* grown in Mueller Hinton Broth (MHB) exhibits a distinct protein profile (7, 8). Interestingly, other investigations have revealed that BHI- and MHB-grown *Ft* also differ in their ability to interact with complement and antibodies (9). In addition, BHI-grown *Ft* exhibits a diminished ability to elicit pro-inflammatory cytokines by various APCs compared with inactivated *Ft* (i*Ft*)-MHB (10, 11). These features of BHI- versus MHB-grown *Ft* provide unique and necessary tools to investigate the dichotomy between the antigenic repertoire versus APC-activating components of whole cell-based *Ft* vaccines.

In this study, we examined the differences in protective efficacy of i*Ft* vaccine prepared by growth in MHB or BHI and further evaluated if the difference in protective efficacy observed reflects changes in not only the immune response but also APC function. While we initially hypothesized that BHI-grown i*Ft*, which more closely mimics the antigenic repertoire of *in vivo* macrophage-grown *Ft*, would provide the superior vaccine immunogen, we found that i*Ft*-MHB generates superior protection compared to i*Ft-*BHI. Other indicators of protection, such as bacterial burden, tissue damage, and production of pro-inflammatory cytokines, also correlated with the improved protective efficacy of i*Ft*-MHB versus i*Ft*-BHI. Furthermore, we found that i*Ft*-MHB exhibited significantly higher binding to APCs, higher induction of dendritic cell (DC) maturation, and increased Ag presentation to *Ft*-specific T cells. Overall, this investigation suggests that APC activation components are important for protective efficacy of inactivated whole cell-based vaccines. Moreover, growth conditions employed to prepare these vaccines can significantly influence their immunological and protective efficacy.

## Materials and Methods

### Mice

Six- to 8-week-old male and female C57Bl/6 mice were obtained from Taconic laboratories (Hudson, NY, USA). Mice were housed in the animal resource facility at Albany Medical Center. Food and water were provided *ad libitum* during the entirety of experiments. Animal studies were reviewed and approved by Institutional Animal Care and Use Committee of Albany Medical Center according to NIH guidelines.

### Preparation of i*Ft*

*Francisella tularensis* LVS was grown in MHB (Becton, Dickinson, Franklin Lakes, NJ, USA) or BHI (Becton, Dickinson) medium at 37°C to a density of ~0.5–1 × 10^9^ colony-forming units (CFU)/ml. To inactivate *Ft* LVS, 1 × 10^10^ CFU/ml of live bacteria were incubated in 1 ml of sterile phosphate-buffered saline (PBS) (Sigma-Aldrich, St. Louis, MO, USA) containing 2% paraformaldehyde (Sigma-Aldrich) for 2 h at room temperature with gentle shaking. Subsequently, fixed bacteria (i*Ft*) were washed with sterile PBS three times. Inactivation was verified by culturing a 100 µl of sample (1 × 10^9^ i*Ft* organisms) on chocolate agar plates (Becton, Dickinson) for 7 days. Protein contents were quantified using a bicinchoninic acid assay (Sigma-Aldrich) as described (12). The i*Ft* preparations were resuspended in PBS and stored in 1 ml aliquots at −20°C until use. *Ft* LVS for challenge was grown similarly and stored in liquid nitrogen until use.

### Immunization and Challenge

Groups of six to eight mice with equal numbers of males and females were immunized on day 0 and 21 *via* the intranasal (i.n.) route. Each mouse was anesthetized by intraperitoneal injection of 20% ketamine plus 5% xylazine and subsequently administered 20 µl of PBS (vehicle), i*Ft*-MHB, or i*Ft*-BHI at a concentration of 37.5 ng, 75 ng, 150 ng, or 300 ng/20 μl. Immunized mice were then challenged on day 35 i.n. with 2× LD_50_ of *Ft* in 40 µl of PBS (single nostril) and subsequently monitored for survival for 21 days.

### Quantification of Bacterial Burden

Following immunization and challenge, mice were euthanized at various time intervals, and lung and spleen were collected aseptically in PBS-containing protease inhibitor mixture (Roche Diagnostics, Indianapolis, IN, USA) and subjected to mechanical homogenization using Mini Bead Beater-8 (BioSpec Products, Bartlesville, OK, USA) using 1-mm zirconia/silica beads. Tissue homogenates were then diluted 10-fold in sterile saline, and 10 µl of each dilution was spotted onto chocolate agar plates in duplicate and incubated at 37°C for 48 to 72 h. The number of colonies on the plates were counted and expressed as CFU per milliliter for the respective tissue. Tissue homogenates were then centrifuged at 14,000 g for 20 min, and the clarified homogenates were stored at −20°C for subsequent cytokine analysis.

### Lactate Dehydrogenase (LDH) Assay

Serum LDH levels were quantified following the manufacturer’s instructions (Sigma-Aldrich). Briefly, mice were immunized and challenged as described for quantification of bacterial burden. Sera were collected on days 1, 3, 5, and 7 postinfection. Prechallenge sera were collected 11 days after booster immunization. Serum samples were diluted 1:100 with LDH assay buffer. Fifty microliters of diluted serum samples, standard, and blanks was added to separate wells in a 96-well microtiter plate. Fifty microliters of LDH substrate was subsequently added to each well. LDH converts NAD to NADH, which can be read calorimetrically at 450 nm. The amount of NADH generated between T_initial_ (absorbance at 450 nm immediately after the LDH substrate) and T_final_ (absorbance at 450 nm immediately after the LDH substrate) by the LDH activity is first calculated (*B*). The LDH activity of the sample is determined by the formula LDH activity = (*B* × sample dilution factor)/(reaction time × *V*), where *V* is the sample volume added to well. LDH activity is reported as milliunit per milliliter.

### Antibody (Ab) Titer Determination

Anti-*Ft-*Ab production in response to immunization with i*Ft*-MHB versus i*Ft*- BHI was measured by ELISA. Briefly, ELISA plates (Corning, Corning, NY, USA) were coated with 50 µl of *Ft* LVS (5 × 10^7^ CFU/ml) in carbonate buffer [4.3 g/l sodium bicarbonate (Sigma-Aldrich) and 5.3 g/l sodium carbonate (Sigma-Aldrich) at pH 9.4] for 16 h at 4°C. The plates were then washed with washing buffer [PBS (Sigma-Aldrich) containing 0.5% bovine serum albumin (BSA) (Sigma-Aldrich)] and blocked for 2 h with 200 µl of PBS containing 5% BSA. Serial threefold dilutions of sera (starting with 1/10) were added to the plates (100 µl/well) and incubated for 2 h at 4°C. After three washes with washing buffer, alkaline phosphatase-conjugated anti-mouse Ab specific for IgG (Sigma-Aldrich) was added and incubated for 1 h at 4°C. Plates were then washed three times with washing buffer, and then 100 µl of BCIP/NBT alkaline phosphatase substrate (Sigma-Aldrich) was added. Plates were subsequently incubated for 2 h, and absorbance at 405 nm was measured using a microplate reader (Molecular Devices, Sunnyvale, CA, USA).

### Sodium Dodecyl Sulfate Polyacrylamide Gel Electrophoresis (SDS-PAGE) and Western Blot Analysis

Samples derived from 10 µg (~1 × 10^8^ cells) of *Ft* were mixed with Laemmli sample buffer (BioRad, Hercules, CA, USA) and boiled for 10 min prior to resolution through 4–12% gradient SDS-PAGE precast gels (Invitrogen, Carlsbad, CA, USA). The running buffer was NuPAGE MES SDS buffer (Invitrogen). Gels were run at 90–160 V. Resolved gels were stained with either Coomassie blue (Bio-Rad) or transferred to nitrocellulose membranes. Coomassie-stained gels were scanned into Adobe Photoshop. Membranes were then blocked for 30 min with PBS, 0.05% Tween 20, 5% non-fat dry milk. Primary Abs were applied for overnight incubation at dilutions ranging from 1:500 to 1:60,000 in PBS-Tween. Following five washes with PBS-Tween, the membranes were incubated for 1 h with agitation with biotinylated goat anti-mouse IgG (H + L, Southern Biotech, Birmingham, AL, USA). Following an additional five washes with PBS-Tween, the membranes were incubated for 1 h with agitation with streptavidin-conjugated horseradish peroxidase (Southern Biotech). Development of the chemiluminescent substrate (SuperSignal West Pico, Pierce, Rockford, IL, USA) was visualized using an Alpha Innotech imaging system in movie mode. Densitometric analysis of developed blots was performed as previously described (7, 9).

### Ag-Presenting Cells

Dendritic cells were generated from bone marrow cells using mouse recombinant FLT-3 (rFLT-3) (R& D systems, Minneapolis, MN, USA) following a previously described method (13). Briefly, bone marrow cells were obtained from 6- to 8-week-old female C57Bl/6 mice and were incubated for 6 days in bone marrow-derived DC (BMDC) generation medium [Roswell Park Memorial Institute (RPMI) 1640 medium] supplemented with 10% fetal bovine serum (FBS), 50 ng/ml rFLT-3, 1 mM sodium pyruvate (Cellgro, Tewksbury, MA, USA), 2 mM l-glutamine (Cellgro), 1× non-essential amino acids (Cellgro), 0.05 mM 2-Mercaptoethanol (Bio-Rad), and 1× penicillin streptomycin (Cellgro). Fifty percent of the medium was replaced with fresh BMDC generation medium on day 3 during 6 days of incubation. Peritoneal exudate cells (PECs) were isolated from naïve mice. PECs were cultured with RPMI 1640 supplemented with 10% FBS, and non-adherent cells were removed by aspiration of medium after 24 h. Adherent cells, which comprise >90% macrophages, were used as APCs.

### i*Ft* Binding Assay

Binding of i*Ft* was visualized by flow cytometry using GFP-expressing i*Ft*, as previously described (13). GFP-expressing *Ft* LVS organisms were provided by Dr. Mats Forsman (Swedish Defense Research Agency, Stockholm) (14). Briefly, 1 × 10^6^ BMDCs were incubated with 5, 10, 15, or 20 µg of GFP-expressing i*Ft*-BHI or i*Ft*-MHB in PBS. After 2 h incubation at 4°C, BMDCs were washed twice with PBS and stained with rat anti-mouse CD11c-Alexa 700 Ab and subsequently analyzed by flow cytometry (LSRII, BD, Franklin Lakes, NJ, USA). The percent of GFP-positive CD11c DCs (indicative of i*Ft* bound to DCs) were analyzed in respective samples.

### *In Vitro* Analysis of Costimulatory Molecule Expression and Cytokine Secretion

Expression of costimulatory molecules on BMDCs was analyzed by flow cytometry as previously described (13). Briefly, 2 × 10^5^ BMDCs were incubated for 24 h with PBS or 10 µg of i*Ft*- MHB or i*Ft*- BHI in an equivalent volume of PBS. Cells were then harvested and stained with Abs to the following surface markers: rat anti-mouse CD40-PE, rat anti-mouse CD80-APC, and rat anti-mouse CD86-Alexa 700. Cells were subsequently analyzed *via* flow cytometry.

Cytokine secretion by BMDCs was assayed following previously described methods (13). Briefly, 2 × 10^5^ BMDCs were incubated with PBS or with 10 µg i*Ft-* MHB or i*Ft-*BHI for 24 h at 37°C. Culture supernatants were then collected and analyzed for cytokines using the Bio-Plex assay system as described below.

### Cytokine Analysis

Cytokines were measured using Bio-Plex assay kits (Bio-Rad) following the manufacturer’s instructions. Briefly, 50 µl of a 1× mixture of magnetic beads coupled with capture Abs for capturing desired cytokines were added to separate wells of flat bottom 96-well microtiter plate. Beads were washed (2×) with Bio-Plex wash buffer. Unless stated otherwise, all incubations were done at room temperature with plates sealed with a plastic sealer and covered with aluminum foil with gentle shaking. All washing was done with Bio-Plex washing buffer. Fifty microliters of diluted standards, blank, or samples was added to individual wells. Plates were incubated for 30 min and washed 3× with Bio-Plex washing buffer. Then 25 µl of 1× mixture of biotinylated detection Abs were added to each well and incubated for 30 min. Plates were washed again, and 50 µl of phycoerythrin-coated streptavidin was added to each well and incubated for 10 min. Plates were then rewashed, and 125 µl of assay buffer was added to each well. Plates were read *via* Luminex reader (Bio-Rad), and the quantity of each cytokine was deduced using a standard curve included in the assay.

### Ag Presentation Assays

Antigen presentation assays were done as previously described (13). Briefly, 2 × 10^5^ BMDCs or PECs were incubated overnight at 37°C with PBS or 5 µg of i*Ft*-MHB or i*Ft*-BHI. A total of 1 × 10^5^ *Ft*-specific T cells (FT25 6D10 hybridoma cells) (13) in 100 µl medium (RPMI 1640 supplemented with 10% FBS) were then added to each well. The plate was then incubated at 37°C for 24 h in CO_2_ incubator, and supernatants were collected. Supernatants were then assayed for interleukin (IL)-5 (secreted by these cells in response to Ag) using the Bio-Plex assay as described earlier.

### Statistics

The log-rank (Mantel-Cox) test was used for survival curves. One-way analysis of variance or the unpaired Student’s *t*-test was used for the remaining figures. Data analysis was performed using Graph-Pad Prizm 5 (Graph-Pad Software, San Diego, CA, USA).

## Results

### i*Ft* Exhibits Protein Profiles Similar to Its Live Counterpart

**Figure 1:**
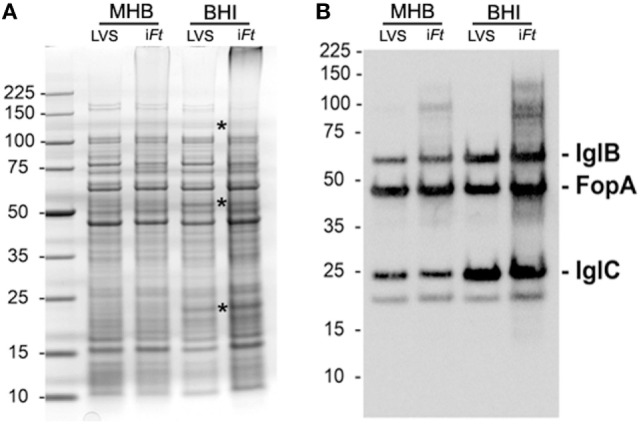
**Fixed *Francisella tularensis* (*Ft*) LVS used herein for immunization studies retain the protein composition of their unfixed counter parts**. Ten micrograms of live and fixed *Ft* LVS previously grown in Mueller Hinton Broth and Brain-Heart Infusion (BHI) were resolved by sodium dodecyl sulfate polyacrylamide gel electrophoresis and stained with Coomassie blue **(A)** or transferred for western blot analysis **(B)**. Asterisks in A mark bands, which are visibly more abundant in the western blot of BHI-grown *Ft* **(B)**. The membrane was probed with a cocktail of monoclonal antibodies specific for IglB, IglC, and a FopA-specific polyclonal mouse serum. IglB and IglC have previously been shown to be more abundant in BHI-grown *Ft* (7, 9). FopA is used here as a loading control. The ~18-kDa band visible under IglC is an unidentified, endogenously biotinylated *Ft* protein (likely AccB) detected by the streptavidin–horseradish peroxidase conjugate used during development of the western blots.

Before using i*Ft* as immunogen, we first used SDS-PAGE and western blot analysis to examine the impact of our fixation procedure on the protein composition of *Ft* LVS. The overall protein profile of live and fixed *Ft* LVS was largely unchanged, although the fixed samples did show some expected evidence of cross-linked Ags (Figure 1A). Consistent with previous reports (7, 9), the BHI-derived samples contained elevated levels of ~23 and ~55 kDa proteins [Figure 1A (asterisks)], which were apparent in both the live and fixed bacteria. Western blot analysis indicated that the live and fixed BHI-derived samples contained levels of IglB and IglC that were elevated relative to their MHB-grown counterparts (Figure 1B). Densitometric analysis revealed that these increases were on the order of threefold to fivefold (Table S1 in Supplementary Material), as reported previously (7, 9). As our fixed i*Ft* preparations appeared to be faithful representations of their live counterparts, we next examined the vaccine efficacy of these immunogens.

### i*Ft-*MHB Generates Superior Protection against *Ft* LVS-Induced Mortality versus i*Ft*-BHI

**Figure 2:**
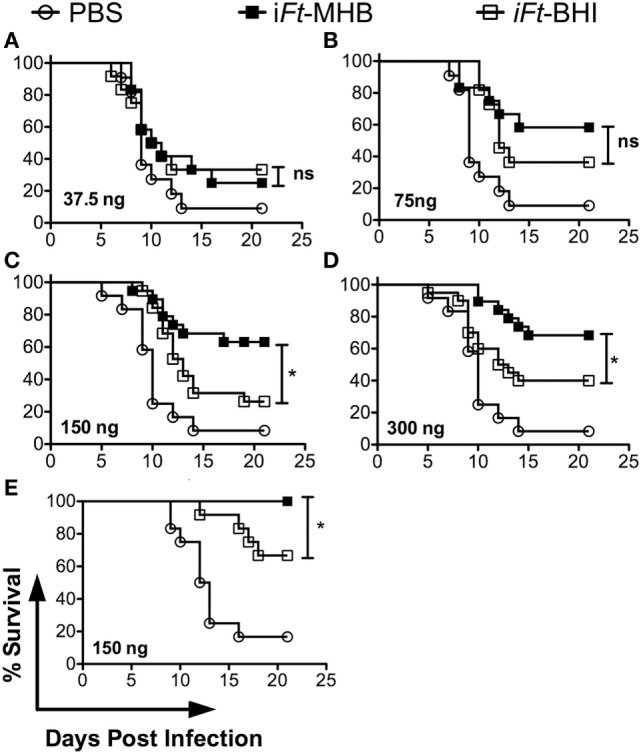
**Inactivated *Francisella tularensis* (i*Ft*)-Mueller Hinton Broth (MHB) provides superior protection compared to i*Ft*-Brain-Heart Infusion (BHI) against lethal *Ft* LVS challenge**. Mice were immunized twice on days 0 and 21 with phosphate-buffered saline or i*Ft*-MHB or i*Ft*-BHI at a dose of 37.5 ng **(A)**, 75 ng **(B)**, 150 ng **(C,E)**, or 300 ng **(D)**. **(A–D)** Mice were infected 2 weeks after booster immunization with 2× LD_50_ of *Ft* LVS grown in MHB. **(E)** Mice were infected with 2× LD_50_ of *Ft* LVS grown in BHI. This figure represents Kaplan–Meier survival curves of three independent experiments with a total of 20 mice per group **(A–D)** or 1 experiment with 12 mice per group **(E)**. *p < 0.05.

First, we evaluated whether the differential expression of antigenic repertoire and PAMPs affected by cultivation in MHB versus BHI has any impact on the protective efficacy of i*Ft*. We used i*Ft* generated in MHB or BHI to immunize mice. Following a lethal challenge with *Ft* LVS, we observed that i*Ft*- MHB-immunized mice were better protected than i*Ft*-BHI-immunized mice (Figure 2). Of the four doses used, the 150- and 300-ng doses exhibited statistically significant differences in the protection afforded by i*Ft*-MHB versus i*Ft*-BHI (Figures 2C,D). Importantly, in Figures 2A–D mice were challenged with MHB grown *Ft* LVS. Thus, to evaluate any potential bias of growth medium used to culture the challenge organism (*Ft* LVS) on the outcome of survival, we similarly immunized mice with PBS or 150 ng of i*Ft*-MHB or i*Ft*-BHI, but challenged with BHI-grown *Ft* LVS Once again, we observed that i*Ft*-MHB provided better protection compared to i*Ft*-BHI (Figure 2E).

### i*Ft-*MHB-Immunized Mice Are Better Protected against Bacterial Replication and Tissue Damage following *Ft* LVS Infection, as Compared to That of i*Ft*-BHI

**Figure 3:**
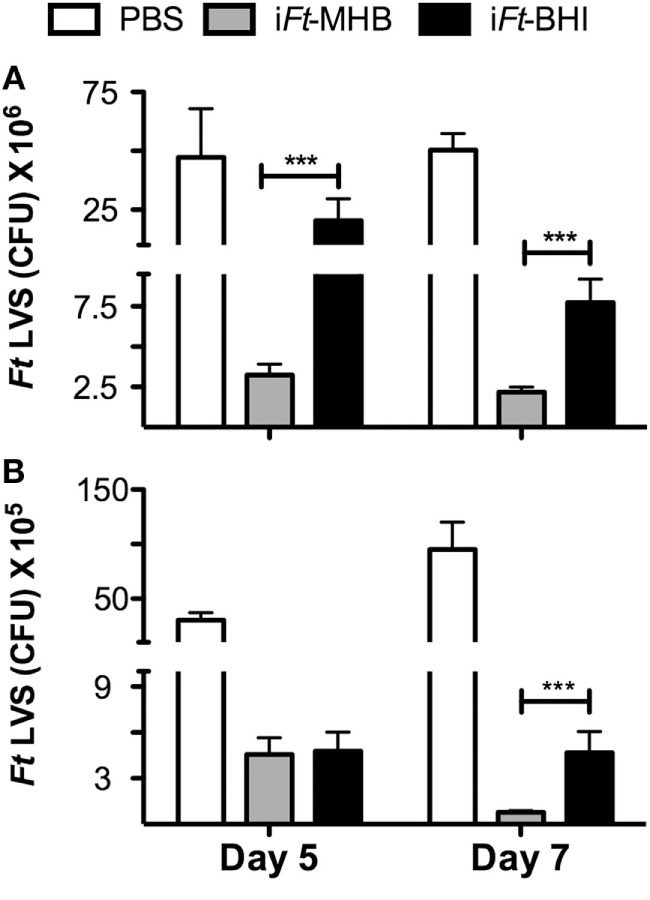
**Inactivated *Francisella tularensis* (i*Ft*)-Mueller Hinton Broth (MHB) better reduces bacterial burden following *Ft* LVS infection as compared to that of i*Ft*-BHI: mice were immunized as in Figure 2 with phosphate-buffered saline (white bars), i*Ft*-MHB (gray bars), or i*Ft*-BHI (black bars) and challenged with *Ft* LVS grown in MHB**. Subsequently, *Ft* LVS colony-forming units were enumerated in the lung **(A)** and spleen **(B)** homogenates at indicated days postinfection. Each bar represents the mean ± SE (error bar) of two independent experiments with a total of eight mice per group. ***p < 0.001.

Following the above observation of differential protection afforded by i*Ft*-MHB versus i*Ft*-BHI, we investigated the ability of the two i*Ft* immunogens to protect against bacterial replication and tissue damage exerted by *Ft* LVS infection. Following immunization and *Ft* LVS challenge, we found significantly lower bacterial burdens in the lungs (Days 5 and 7) (Figure 3A) and spleen (Day 7) (Figure 3B) of mice immunized with i*Ft*-MHB as compared to that of i*Ft*-BHI.

**Figure 4:**
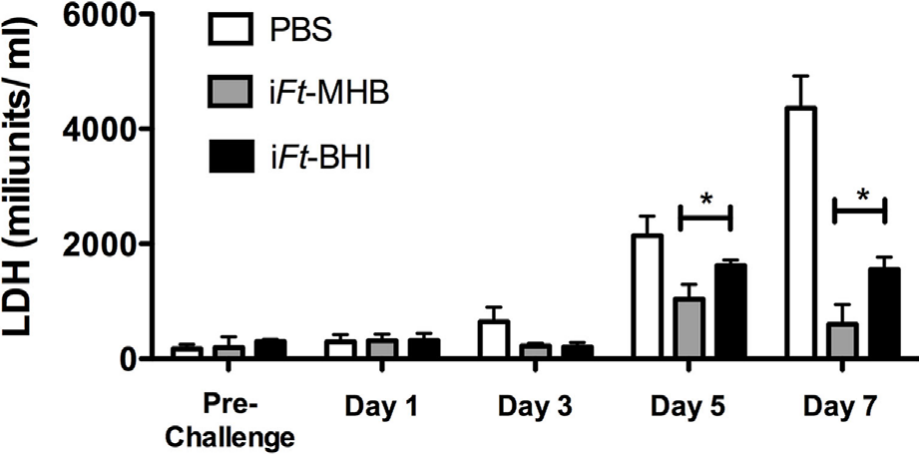
**Inactivated *Francisella tularensis* (i*Ft*)-Mueller Hinton Broth (MHB) induces superior resistance to *Ft* LVS-induced systemic tissue damage compared to that of i*Ft*-Brain-Heart Infusion (BHI): mice were immunized on days 0 and 21 with phosphate-buffered saline (white bars), i*Ft*-MHB (gray bars), or i*Ft*-BHI (black bars) and challenged with *Ft* LVS grown in MHB**. Lactate dehydrogenase activity was quantified in serum drawn on indicated days postchallenge. Prechallenge sera were drawn 11 days after boost. Each bar represents mean ± SE (error bar) of two independent experiments with a total of eight mice per group. *p < 0.05.

*Francisella tularensis* LVS infection inflicts systemic tissue damage in infected mice, and serum LDH levels are commonly used as a biomarker of tissue damage (15). Thus, we evaluated serum LDH levels at early and late stages of *Ft* LVS infection in mice immunized with i*Ft*-MHB versus i*Ft*-BHI. At days 5 and 7 after *Ft* LVS infection, significantly lower levels of LDH activity were found in i*Ft*-MHB-immunized mice versus that of i*Ft*-BHI-immunized mice (Figure 4).

### Differential Protection against *Ft* LVS-Induced Inflammation by i*Ft*-MHB versus i*Ft*-BHI

**Figure 5:**
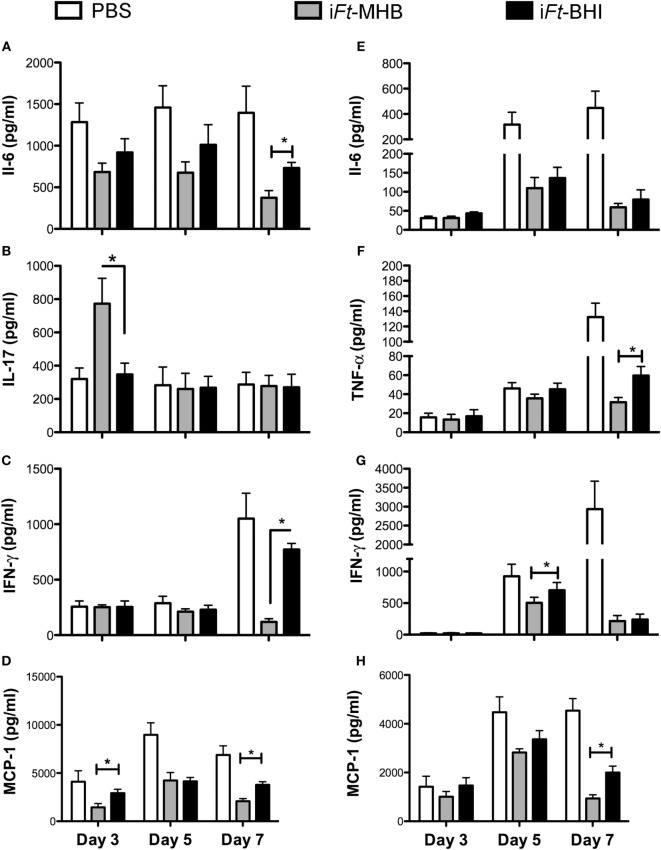
**Inactivated *Francisella tularensis* (i*Ft*)-Mueller Hinton Broth (MHB) is superior to i*Ft*-Brain-Heart Infusion (BHI) in controlling *Ft*-induced inflammation in the lung**. Mice were immunized with phosphate-buffered saline (white bars) or 150 ng of i*Ft*-MHB (gary bars) or i*Ft*-BHI (black bars) and challenged with *Ft* LVS Indicated cytokines were quantified in homogenates of lung **(A–D)** and spleen **(E–H)** at the indicated days postinfection. Values represent mean ± SE (error bar) of two independent experiments with a total of eight mice per group. *p < 0.05.

Systemic inflammation and/or sepsis are hallmarks of the tularemia in advanced stages of infection. We previously observed that mice protected by immunization exhibit lower levels of pro-inflammatory cytokines in the lungs and spleens following *Ft* infection, compared to those of unprotected mice (16, 17). In this case, analogous to our previous studies, we observed that mice immunized with i*Ft*-MHB, which are better protected, also exhibited lower levels of IL-6 (Figure 5A), interferon (IFN)-γ (Figure 5C), and monocyte chemoattractant protein (MCP)-1 (Figure 5D) in the lungs in the latter stage of disease (day 7 postinfection). MCP-1 levels were also lower at day 3 postinfection in the lungs of i*Ft*-MHB immunized mice compared to that of i*Ft*-BHI immunized mice. MCP-1 is a chemoattractant for inflammatory monocytes, which can induce to tissue damage. Therefore, regulation of MCP-1 levels is important for protection against systemic tissue damage and subsequent mortality inflicted by *Ft* LVS infection. Interestingly, higher levels of IL-17 were also found at day 3 postinfection in i*Ft*-MHB immunized mice compared to that of i*Ft*-BHI immunized (Figure 5B). IL-17 has been found to be protective against mucosal infections with bacteria including *Ft* (18, 19). Thus, higher IL-17 levels at early stages of infection correlate with superior protection afforded by i*Ft*-MHB compared to i*Ft*-BHI. Similarly, in the spleens, significantly lower levels of tumor necrosis factor (TNF)-α (at day 7 postinfection) (Figure 5F), IFN-γ (day 5 postinfection) (Figure 5G), and MCP-1 (day 7 postinfection) (Figure 5H) were observed in mice immunized with i*Ft*-MHB compared to that of i*Ft*-BHI. However, level of IL6 (Figure 5E) in the spleens were comparable among mice immunized with i*Ft*-MHB and i*Ft*-BHI.

### Increased Ab Responses Are Induced by i*Ft*-MHB versus i*Ft*-BHI

**Figure 6:**
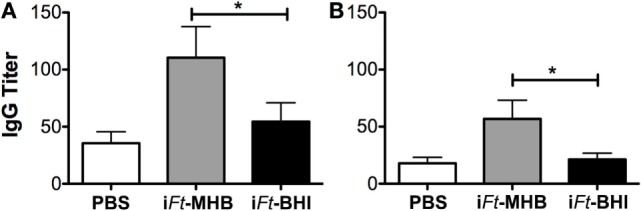
**Increased *Francisella tularensis* (*Ft*)-specific antibody production is induced by inactivated *Ft* (i*Ft*)-Mueller Hinton Broth (MHB) versus i*Ft*-Brain-Heart Infusion (BHI)**. Mice were immunized twice at a 3-week interval with 20 µl phosphate-buffered saline (PBS) or 150 ng of i*Ft*-BHI or i*Ft*-MHB *via* the intranasal route in 20 µl PBS. Sera obtained 2 weeks postboost were analyzed for *Ft*-MHB-specific **(A)** and *Ft*-BHI-specific **(B)** IgG. Values represent mean ± SE of two independent experiments (*n* = 16 mice per group). *p < 0.05.

Next we investigated whether i*Ft* generated in MHB versus BHI medium also has a differential impact on the humoral immune response against *Ft*. We quantified *Ft-*specific serum-IgG by ELISA as a measure of humoral immune response to *Ft*. Live *Ft* LVS grown in BHI and MHB medium were coated onto ELISA plates and reacted with sera obtained from i*Ft*-MHB versus i*Ft*-BHI-immunized mice. IgG induced by i*Ft*-MHB compared to i*Ft*-BHI reacted more strongly to *Ft* surface Ags expressed on both MHB- or BHI-grown *Ft* LVS, as evidenced by their relative titers (Figures 6A,B). This is consistent with superior protection afforded by i*Ft*-MHB compared to that of i*Ft*-BHI. The choice of Ags employed [*Ft*-MHB (Figure 6A) versus *Ft*-BHI (Figure 6B)] to detect Ab titers did not alter the relative pattern of Ab production observed in i*Ft*-MHB or i*Ft*-BHI-immunized mice.

**Figure 7:**
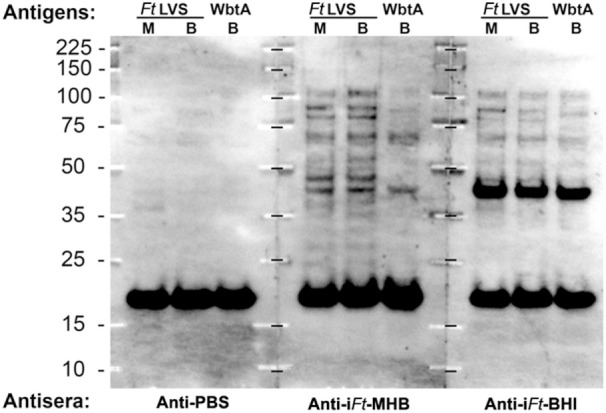
**Inactivated *Francisella tularensis* (i*Ft*)-Mueller Hinton Broth (MHB) elicits antibody (Ab) against broader range of antigens compared to i*Ft*-Brain-Heart Infusion (BHI)**. Ten micrograms of *Ft* LVS or WbtA LVS grown as indicated (M = MHB, B = BHI) were resolved by sodium dodecyl sulfate polyacrylamide gel electrophoresis and transferred to nitrocellulose membranes for western blot analysis. Membranes were incubated overnight with 1:500 dilutions of the indicated sera. Bound Abs were detected through sequential incubations with biotinylated goat anti-mouse and streptavidin-conjugated horseradish peroxidase (HRP). The prominent ~18 kDa band visible in all three blots is an unidentified, endogenously biotinylated *Ft* protein detected by the SA-HRP conjugate.

To further characterize these Ab responses, we also used the above sera to probe by western blot lysates of MHB- and BHI-grown *Ft* LVS along with a BHI-grown mutant *Ft* (WbtA), which is deficient for O-Ag. As shown in Figure 7, the anti-i*Ft*-MHB sera recognized a more diverse array of Ags than did the anti-i*Ft*-BHI sera. One of the Ags preferentially recognized by the anti-i*Ft-*MHB sera appeared to have a ladder pattern typical of LPS, and consistent with this, this ladder pattern was markedly reduced/absent in the WbtA mutant *Ft*. In addition, the Ag specificity of the anti-i*F-*BHI sera appeared to be more focused on a ~43 kDa protein with reduced O-Ag-dependent reactivity. Collectively, the data in Figures 6 and 7, as well as in data not shown, indicate that immunization with i*Ft*-MHB induces higher titers of anti-*Ft* Ab and that a larger proportion of this Ab is dependent on O-Ag for binding.

### i*Ft*-MHB and i*Ft*-BHI Differentially Bind to BMDCs and Differentially Induce Expression of Costimulatory Molecules

Having observed differential protective efficacy between i*Ft* generated in MHB versus BHI, we sought to further identify the immune mechanisms underlying the differences in protective efficacy of i*Ft*-MHB versus i*Ft*-BHI. In our previous studies (7, 9) and Figure 1, we demonstrated that i*Ft*-BHI exhibits an antigenic profile more similar to that of *in vivo* circulating *Ft*, as apposed to that of *Ft*-MHB. This is in contrast to our expectation that by virtue of exposing the host to i*Ft*-BHI, which is more similar antigenically to *in vivo* replicating *Ft*, a better protective immune response would be achieved. This led us to further investigate the immune mechanisms responsible for this unexpected observation.

**Figure 8:**
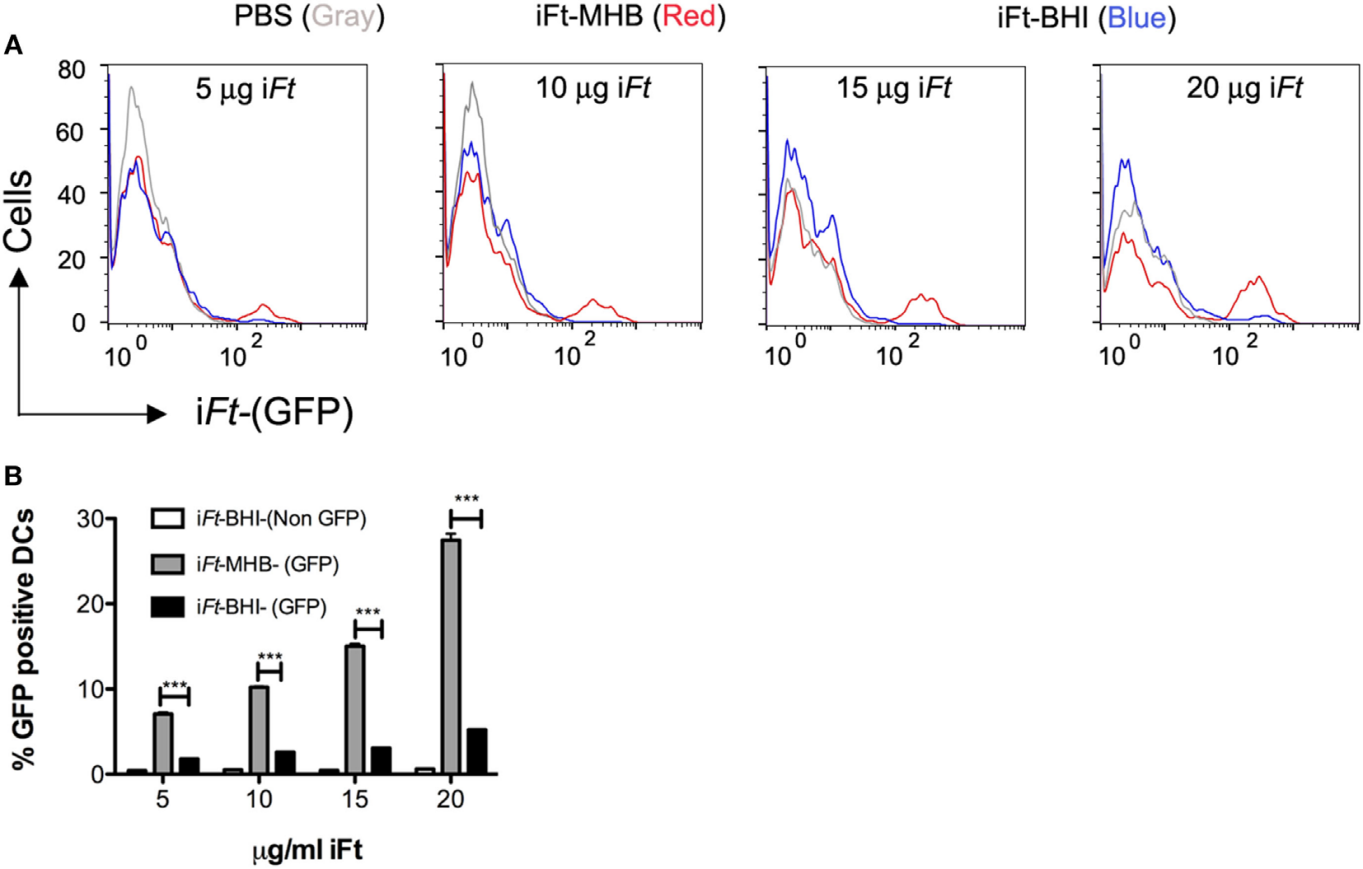
**Enhanced binding of inactivated *Francisella tularensis* (i*Ft*)-Mueller Hinton Broth (MHB) to antigen-presenting cells (APCs) as compared to i*Ft*-Brain-Heart Infusion (BHI)**. Bone marrow-derived dendritic cells (BMDCs) were incubated with GFP-expressing i*Ft*-BHI or i*Ft*-MHB. Cells were washed and stained with CD11c antibody before analyzing with flow cytometry for GFP signal associated with BMDCs **(A)**. **(B)** Percentage of GFP-positive (+) BMDCs as evaluated by flow cytometry. Values represent mean ± SE of two independent experiments. ***p < 0.001.

**Figure 9:**
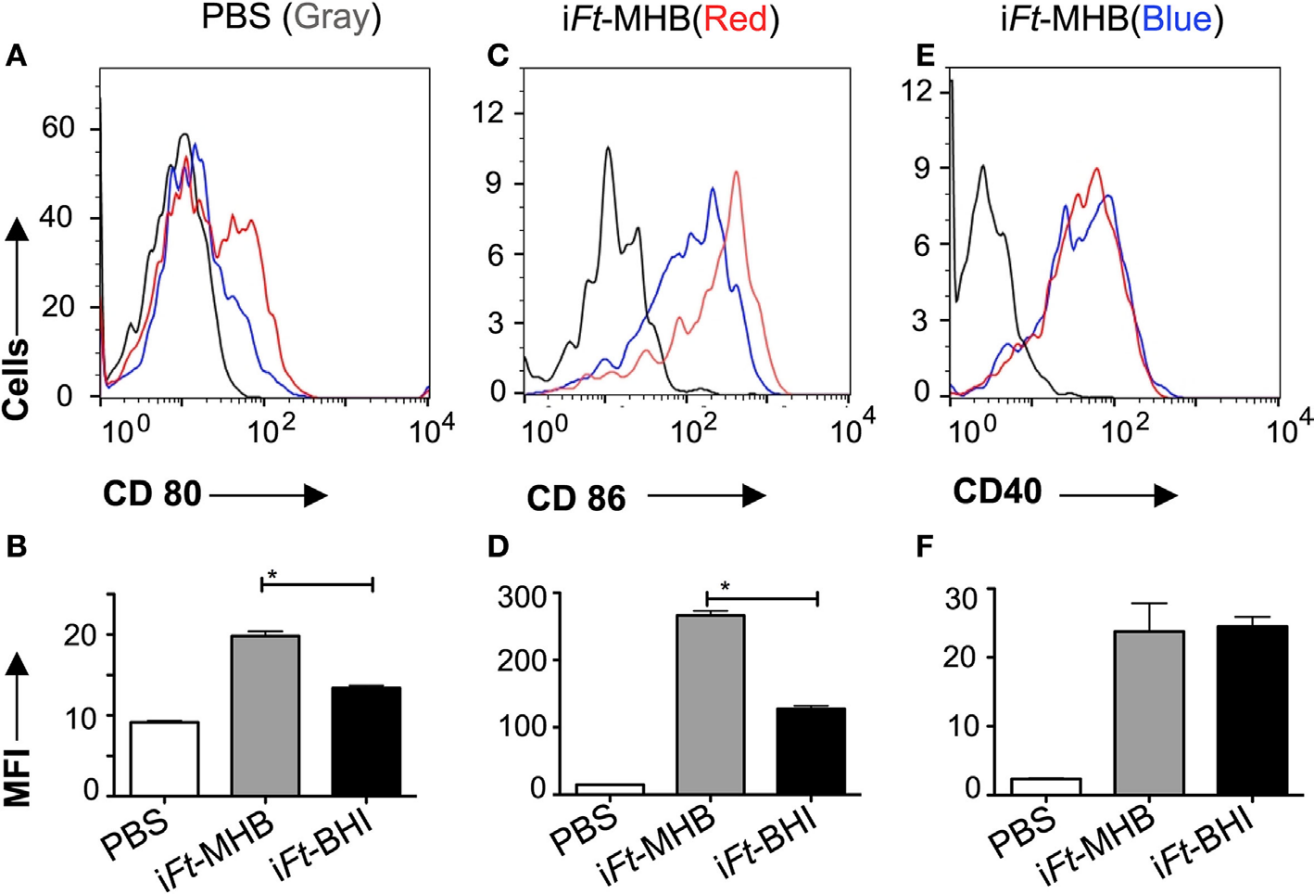
**Induction of higher expression of costimulatory molecules on antigen-presenting cells by inactivated *Francisella tularensis* (i*Ft*)-Mueller Hinton Broth compared to i*Ft*-Brain-Heart Infusion (BHI)**. Bone marrow-derived dendritic cells were incubated with i*Ft*-BHI and i*Ft*-MHB for 24 h. Expression of indicated cell surface markers were analyzed *via* flow cytometry **(A,C,E)**. **(B,D,F)** The MFI of respective markers. Values represent mean ± SE of two independent experiments. *p < 0.001.

By using a GFP-expressing *Ft* LVS, we investigated whether i*Ft* generated in MHB versus BHI have different abilities to bind to and activate APCs. In fact, we observed significantly higher binding of i*Ft*-MHB-(GFP) to BMDCs compared to i*Ft*-BHI-(GFP) (Figure 8). Increasing the concentration of the i*Ft*-MHB-(GFP) successively increased the number of BMDCs harboring i*Ft*-MHB. In contrast, i*Ft*-BHI-(GFP) exhibited relatively poor binding to BMDCs at all concentrations used in this assay. Furthermore, costimulatory molecules are essential components for T cell activation. As demonstrated in Figure 9, i*Ft*-MHB induced significantly higher expression of the costimulatory molecules CD80 (Figures 9A,B) and CD86 (Figures 9C,D), as compared to i*Ft*-BHI, although CD40 (Figures 9E,F) expression was stimulated to a similar degree by both immunogens.

### i*Ft*-MHB Mediates Higher Pro-inflammatory Cytokine Production and Increased Ag Presentation to *Ft*-Specific T Cells

**Figure 10:**
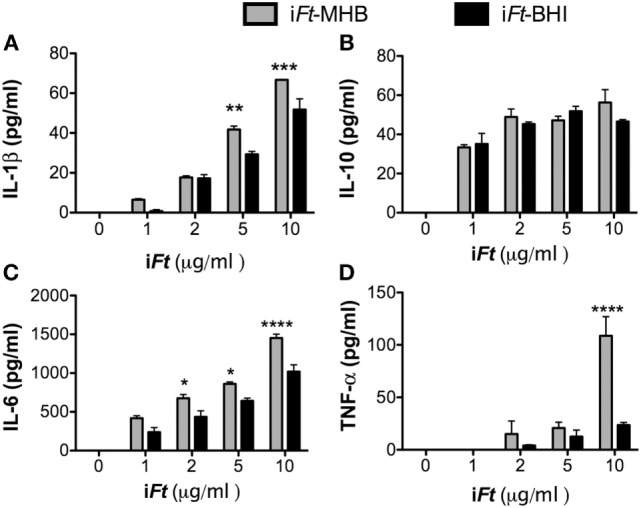
**Inactivated *Francisella tularensis* (i*Ft*)-Mueller Hinton Broth (MHB) elicits greater pro-inflammatory cytokine production compared to i*Ft*-Brain-Heart Infusion (BHI)**. Bone marrow-derived dendritic cells were incubated with phosphate-buffered saline, i*Ft*-BHI, or i*Ft*-MHB for 24 h. Supernatants were analyzed for **(A)** IL1B, **(B)** IL10, **(C)** IL6, and **(D)** TNF-α. Values presented are mean ± SE of two independent experiments. *p < 0.05, **p < 0.01, ***p < 0.001, and ****p < 0.0001.

**Figure 11:**
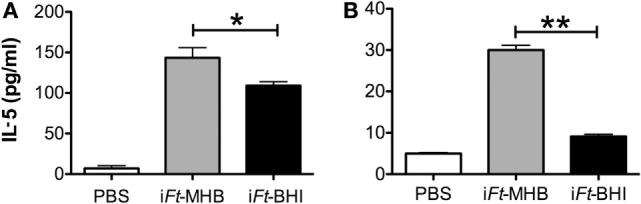
**Relative presentation of inactivated *Francisella tularensis* (i*Ft*)-Brain-Heart Infusion (BHI) and i*Ft*-Mueller Hinton Broth (MHB) by antigen-presenting cells (APCs): APCs [peritoneal exudate cells (A) or bone marrow-derived dendritic cells (B)] were exposed to phosphate-buffered saline or 10 µg of i*Ft*-MHB or i*Ft*-BHI for 24 h**. FT25 6D10 cells were then mixed with the APCs and incubated for 24 h. Supernatants were then collected analyzed for interleukin-5. Values represent mean ± SE of two independent experiments. *p < 0.05, **p < 0.01.

In addition to Ag binding and expression of costimulatory molecules on APCs, secretion of pro-inflammatory cytokines is also critical for DC-mediated orchestration of the adaptive immune response. We observed that i*Ft*-MHB induces higher levels of the pro-inflammatory cytokines [IL-1β (Figure 10A), IL-6 (Figure 10C), and TNF-α (Figure 10D)], as compared to i*Ft*-BHI, while both immunogens induced similar levels of IL-10. Finally, we investigated the presentation of i*Ft*-MHB versus i*Ft*-BHI using an *Ft-*specific T cell hybridoma (FT25 6D10), which is specific to an epitope corresponding to *Ft* 50 s-ribosomal protein (20) and secretes IL-5 on engagement with cognate major histocompatibility complex (MHC)-peptide complex (13). Co-cultures of FT25 6D10 and APCs (PECs or BMDCs) exposed to i*Ft*-MHB exhibited higher levels of IL-5 compared to that of i*Ft*-BHI (Figure 11). Together, data presented in Figures 8–10 suggest that i*Ft*-MHB harbors a superior ability to stimulate APC function as compared to i*Ft*-BHI.

## Discussion

In the present investigation, we observed that i*Ft* propagated in MHB versus BHI medium exhibits a differential ability to stimulate an immune response to and protection against *Ft* challenge. Specifically, i*Ft*-MHB immunogen induced improved protection against lethal *Ft* LVS challenge as compared to i*Ft-*BHI. Consistent with the superior protective activity of i*Ft*-MHB immunogen, bacterial burden and tissue damage were also reduced. Furthermore, immunization with i*Ft*-MHB leads to reduced inflammation in the lungs and spleens. Importantly, it has been demonstrated that mice protected against *Ft* LVS challenge *via* immunization exhibit lower pro-inflammatory cytokines in their lungs and spleen late in infection (16, 17), which we also observed in the case of i*Ft*-MHB immunization. Notably, in contrast to the above, however, we observed elevated levels of IL-17 in lungs of mice immunized with i*Ft*-MHB on day 3 postinfection with *Ft* LVS IL-17 has been implicated in protection against mucosal infections including *Ft* LVS (18, 19). Immunization with i*Ft*-MHB also induced higher levels of *Ft*-specific IgG, which have been shown to play a role in protection against *Ft* LVS (21). Apart from quantitative differences in IgG titers, Abs produced in response to i*Ft*-MHB and i*Ft*-BHI also differed qualitatively. While the dominant reactivity of anti-i*Ft*-BHI was to a 43 kDa protein, the anti-i*Ft*-MHB response was more broadly distributed among a range of Ags including *Ft* LPS (O-Ag). *Ft* LPS has been found to be an important Ag for protection against *Ft* infection (22). Although, at present, we do not know the significance of the differences in the Ab responses elicited by i*Ft*-MHB versus i*Ft*-BHI, further studies in this regard may reveal their impact on protection.

In regard to the mechanisms(s) by which i*Ft*-MHB is a better immunogen, APCs are the gateway to adaptive immune response. APCs present Ags to T cells in association with MHC, which provides the primary signal for T cell activation. Other signals required for T cell activation include costimulatory molecules, e.g., CD80 and CD86 (23–25). T cells that receive signals only *via* TCR by engagement with MHC-peptide complex, without receiving the second signal from costimulatory ligands, are destined to undergo anergy (26). Moreover, the local cytokine niche determines the type of T cell response generated. For example, IL-12 and IFN-γ favor a T helper 1 immune response, while IL-10 and transforming growth factor-β favor a regulatory T cell immune response (27, 28). Furthermore, APCs can be activated to express MHC II, costimulatory molecules, and pro-inflammatory cytokines by exposing them to various pathogen-associated molecular patterns (23). Therefore, it is important that APCs are exposed to vaccine Ags in the context of appropriate APC activation signals (29). In this investigation, i*Ft*s derived from growth in MHB medium exhibited a superior ability to activate APCs versus i*Ft*-BHI. Specifically, MHB-derived i*Ft* exhibited an increased ability to induce CD80 and CD86 on BMDCs. i*Ft*-MHB also induced higher pro-inflammatory cytokine production in BMDCs. Moreover, we also observed higher levels of Ag presentation/T cell activation induced by BMDCs exposed to i*Ft*-MHB versus to i*Ft*-BHI. Notably, i*Ft*-MHB also exhibited comparatively greater binding to BMDCs than i*Ft*-BHI. The increased binding may explain the higher levels of pro-inflammatory cytokine stimulation and Ag presentation by i*Ft*-MHB (Figure 7). Observations from other investigators are consistent with our observations (10, 11) that i*Ft*-MHB can induce higher pro-inflammatory cytokines compared to i*Ft*-BHI. Thus, collectively, it is clear that i*Ft*-MHB exhibits a superior ability to impact the nature and magnitude of adaptive immune response, thereby enhancing its protective efficacy. The apparent differences in APC engagement between i*Ft*-MHB and i*Ft*-BHI may be a reflection of their structural differences. In this regard, we have demonstrated that BHI-grown *Ft* LVS expresses additional layers of capsular polysaccharides, which have been implicated in hindering *Ft*s’ ability to bind with complement and Abs (9). Capsular polysaccharides have also been implicated in masking TLR2 and Tul4 ligands on the surface of *Ft* (9). Furthermore, Singh et al demonstrated that *Ft* grown in MHB exhibited increased engagement of cytosolic DNA sensors, possibly due to structural defects resulting in an increased release of DNA into cytosol after uptake by APCs, thereby inducing AIM 2-dependent cytokine production (11). The LPS of *Ft* LVS has a peculiar structure that minimizes its interaction with TLR4. Also, recently it has been shown that genetic engineering of microbes that express *E. coli* LPS significantly enhances their TLR4 engagement with bone marrow-derived macrophage (30). Thus, in future, one potential means to improve *Ft*s’ ability to engage and activate APCs may be to genetically engineer *Ft* to express modified TLR agonists such as *E. coli* LPS.

It is also important to note that environmental conditions such as temperature, metal ions present, pH, and other media ingredients used for *in vitro* growth can significantly influence antigenic expression by bacterial pathogens (8). Various investigations have demonstrated the significance of growth conditions on bacterial recognition by Abs (1–3). In this investigation, we demonstrate that the two growth media employed for preparation of i*Ft* LVS whole cell vaccines significantly altered the vaccine-induced immune response and protection against cognate pathogen. Recently, BCG vaccine exhibited differential protective efficacy based on the presence or absence of detergents in the growth medium. The latter was associated with differential expression of capsular polysaccharides, the presence of capsule specific Ab, splenic IL-17 and IFN-γ production, and multifunctional CD4+ T cell responses (31). We have also observed differential protective efficacy of BHI- and MHB-grown live *Ft* LVS vaccine, where BHI-grown *Ft* LVS exhibited better protection against lethal infection with *Ft* SchuS4 (32). This contrasting observation may be due to the distinct immunological requirements for protection against *Ft* LVS and *Ft* SchuS4 or live versus inactivated vaccine. Thus, we are further investigating this distinction in our laboratory. Nevertheless, these studies strongly emphasize the critical importance of growth conditions when developing whole cell vaccines.

## Conclusion

**Figure 12:**
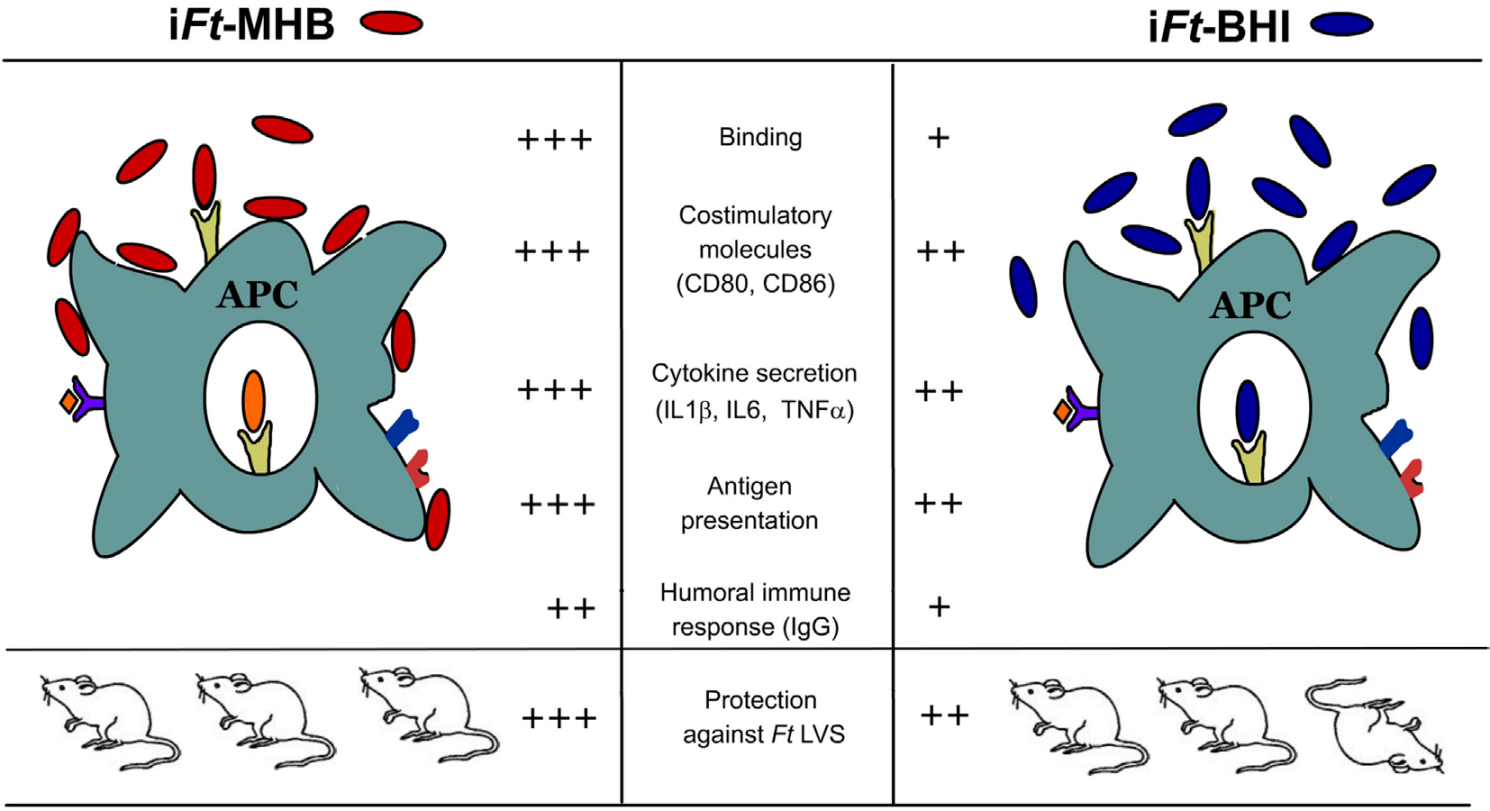
**A model comparing observations when utilizing inactivated *Francisella tularensis* (i*Ft*)-Mueller Hinton Broth (MHB) versus i*Ft*-Brain-Heart Infusion (BHI) as immunogen**. i*Ft*-MHB in comparison to i*Ft*-BHI engages and activates antigen (Ag)-presenting cells (APCs) including significantly higher binding, expression of costimulatory molecules (CD80 and CD86) and secretion of pro-inflammatory cytokines [interleukin (IL)-1b, IL-6, and TNF]. i*Ft*-MHB Ags are also presented to *Ft*-specific T cells more efficiently compared to i*Ft*-BHI. Collectively, superior activation of APCs by i*Ft*-MHB results in higher IgG response and protection against lethal *Ft* challenge.

When we started this investigation, the goal was to improve the efficacy of *Ft* LVS-based whole cell vaccines. Our assumption was that by exposing the host to an antigenic repertoire more similar to that of the infecting agent *in vivo* (i*Ft*-BHI), a superior protective immune response would be generated as compared to i*Ft*-MHB (7). However, as depicted in Figure 12, we observed that i*Ft*-MHB elicited a superior ability to stimulate the immune response and protection, as compared to i*Ft*-BHI. In conclusion, our observations indicate that to endow hosts with protective immune response, vaccine formulations should simultaneously deliver a variety of protective Ags as well as APC activation components. This investigation also emphasizes that *in vitro* growth conditions, which influence Ag expression as well as APC activation capacity, are critical variables that must be considered when developing whole cell-based vaccines.

## Ethics Statement

This study was carried out in accordance with the recommendations of the Institutional Animal Care and Use Committee (IACUC) of Albany Medical Center, Albany, NY, USA. The protocol was approved by the IACUC of Albany Medical Center, Albany, NY, USA.

## Author Contributions

EG, KH, and SK conceptualized and designed the study. SK, RS, GP, BF, and SR performed the experiments and acquired and analyzed the data. SK drafted the manuscript. EG and KH critically revised the manuscript. All the authors approved the publication of the manuscript and agreed to be accountable for all aspects of the work.

## Conflict of Interest Statement

The authors declare that the research was conducted in the absence of any commercial or financial relationships that could be construed as a potential conflict of interest.
