# Supplementary material for: Differential Cultivation of Francisella tularensis Induces Changes in the Immune Response to and Protective Efficacy of Whole Cell-Based Inactivated Vaccines
Source: Front Immunol. 2017 Jan 10;7:677. doi: 10.3389/fimmu.2016.00677 (PMC5222797; doi:10.3389/fimmu.2016.00677)
Supplement: Supplementary file 1 [file Table_1.DOCX]

**Supplemental Table I**

**Densitometric Analysis of Developed Blot Presented in Figure 1B***

| **Protein** | ***Ft*-MHB** | ***Ft*-BHI**** | **i*Ft*-MHB**** | **i*Ft*-BHI**** |
| --- | --- | --- | --- | --- |
|  |  |  |  |  |
| IglB | 1 | 2.8 | 2.1 | 5.1 |
| FopA | 1 | 1.1 | 1.2 | 1.9 |
| IglC | 1 | 2.8 | 0.9 | 3.2 |

* Development of the chemiluminescent substrate (SuperSignal West Pico, Pierce, Rockford, IL) was visualized using an Alpha Innotech imaging system in movie mode. Densitometric analysis of developed blots was performed as previously described (7, 9).

** Values represent fold change over the corresponding measurement in *Ft*-MHB band intensity.
